# Quality Improvement of Few-Layers Defective Graphene from Biomass and Application for H_2_ Generation

**DOI:** 10.3390/nano9060895

**Published:** 2019-06-19

**Authors:** Jinbao He, Aicha Anouar, Ana Primo, Hermenegildo García

**Affiliations:** Instituto Universitario de Tecnología Química, Consejo Superior de Investigaciones Científicas-Universitat Politècnica de Valencia, Av. De los Naranjos s/n, 46022 Valencia, Spain; 2131342@mail.dhu.edu.cn (J.H.); aian1@doctor.upv.es (A.A.)

**Keywords:** defective graphene, chitosan pyrolysis, alginate pyrolysis, graphene from biomass

## Abstract

Pyrolysis of filmogenic natural polymers gives rise to the formation of films of few-layers defective, undoped, and doped graphenes with low electrical conductivity (3000 to 5000 Ω/sq). For the sake of valorization of biomass wastes, it would be of interest to decrease the density of structural defects in order to increase the conductivity of the resulting few-layers graphene samples. In the present study, analytical and spectroscopic evidence is provided showing that by performing the pyrolysis at the optimal temperature (1100 °C), under a low percentage of H_2_, a significant decrease in the density of defects related to the presence of residual oxygen can be achieved. This improvement in the quality of the resulting few-layers defective graphene is reflected in a decrease by a factor of about 3 or 5 for alginic acid and chitosan, respectively, of the electrical resistance. Under optimal conditions, few-layers defective graphene films with a resistance of 1000 Ω /sq were achieved. The electrode made of high-quality graphene prepared at 1100 °C under Ar/H_2_ achieved a H_2_ production of 3.62 µmol with a positive applied bias of 1.1 V under LED illumination for 16 h.

## 1. Introduction

**Scheme 1:**
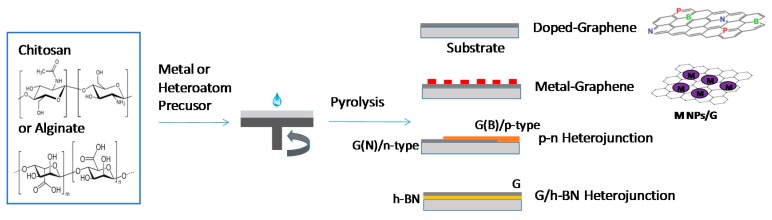
Pictorial illustration of the one-step preparation of doped graphene and various types of graphene heterojunctions.

There is a considerable interest in the development of different graphene preparation methods that, on one hand, can serve to valorize wastes, while at the same time can render materials with adequate properties for a given application [1,2,3,4]. In this context, it was reported that pyrolysis at temperatures above 900 °C of alginic acid and chitosan forms carbon residues that undergo easy exfoliation in high yields to form defective graphenes [5,6,7]. Starting from certain natural *filmogenic* polysaccharides as precursors, pyrolysis can serve for the preparation of defective graphenes either as films or as suspensions [6,7]. This procedure can be adapted also to the preparation of other doped graphenes, either by using a precursor that contains already the heteroatom, like chitosan and λ-carrageenan for the preparation of N- and S-doped defective graphene [7,8], respectively, or alternatively the polysaccharide can be derivatized by reaction with inorganic acids that contain the required heteroatom [9]. Moreover, the method can also be adapted to the one-step preparation of heterojunctions of differently doped defective graphenes, or heterojunctions of graphene with other 2D materials and even to the preparation of graphenes having strongly grafted metal nanoparticles or other graphene heterojunctions with 2D materials (Scheme 1) [10,11,12].

Some of the defects in this type of graphenes derive from the presence of a residual oxygen content, about 20%, remaining from the incomplete carbonization of the polysaccharide precursors that initially contain above 50% oxygen in their composition. Among the various characterization techniques, it has already been indicated that the presence of defects, including oxygenated functional groups, can be easily monitored by Raman spectroscopy and, somehow, quantitatively assessed by the shape and relative intensities of the G vs. the D bands appearing at about 1600 and 1350 cm^−1^, respectively. Typical values of *I*_G_/*I*_D_ in defective graphenes obtained from natural polysaccharide pyrolysis are about 1.15 [7].

Besides Raman spectroscopy, the presence of these defects is typically also reflected in the electrical conductivity of the graphene material. High electrical conductivity is relevant for different applications of graphenes related to microelectronics and to the preparation of transparent and conductive displays, among others [13,14]. In the case of defective graphenes obtained from biomass pyrolysis, it has been reported that the electrical conductivity measured by the four-tips head method on quartz substrates is in the range of a few kΩ/sq, typically from 3 to 10 kΩ/sq, significantly lower than the values reported for ideal graphene on other supports [6].

In this context, considering the added value of the conversion of alginic acid, chitosan, and other natural polysaccharides into graphenes, it would be of interest to improve further the electrical conductivity of these defective graphenes obtained by the pyrolysis of biopolymers by introducing some optimization in the preparation procedure. This could open additional applications for this type of graphene materials. Since oxygen functionalities are one type of defect, it was considered that decrease in the oxygen content of these materials should also be reflected in a diminution of the density of defects, at least those caused by oxygenated functional groups on the resulting graphenes. It should be noted that, in some occasions, oxygen has been used to decompose adsorbents on the graphene sheet and, in this way, it has been used to clean the graphene surface [15,16]. However, in our case, the oxygen is covalently bonded to carbon atoms on the graphene, introducing defects and decreasing graphene electrical conductivity.

Aimed at this purpose, it is well known that the preparation method of graphenes by chemical vapor deposition, as well as some other procedures, are carried out under H_2_ atmosphere [17,18]. During the high temperature pyrolysis, H_2_ can act as chemical reducing agent and it could produce an additional decrease in the oxygen content of the resulting defective graphene by formation of H_2_O or other hydrogenation volatile compounds and, therefore, its presence during the pyrolytic process could be reflected in an improvement of quality of the films obtained in this way that could be accompanied by an increase in the electrical conductivity. However, the details of the influence of the presence of H_2_ during the pyrolysis of natural polysaccharides remain unexplored.

In the present manuscript, it is shown that by carrying out the pyrolysis of alginic acid and chitosan at increasing temperatures in the presence of H_2_ gas, few-layers defective graphenes with lower oxygen content, lower density of defects, and improved electrical conductivity and photoelectric catalytic activity can be obtained. The present study represents a step forward in the direction of valorization of biomass wastes by obtaining high added-value graphene films.

## 2. Materials and Methods

### 2.1. Synthesis of (N)G Films Deposited on Quartz

Chitosan (250 mg) from Aldrich was dissolved in a 12.5 mL aqueous solution. An amount of 250 µL of HOAc solution was added to dissolve chitosan completely. After 2 h under magnetic stirring at room temperature, the solutions were filtered through a syringe of 0.45 µm pore size to remove the impurities present in the commercial chitosan. The films were prepared on a previously cleaned quartz plate (2 × 2 cm^2^) by casting 500 µL of the filtered chitosan solution at 4000 rpm in 45 s. Once dried on a hot plate, the films were pyrolyzed under Ar flow or Ar/H_2_ (5 vol%) or Ar/H_2_ (10 vol%) flow (200 mL min^−1^), increasing the temperature at a rate of 5 °C min^−1^ up to 900, 1000, 1100, and 1200 °C, respectively, and holding the final temperature for 1 h. The sample was allowed to cool at room temperature under inert atmosphere by stopping electrical heating.

### 2.2. Synthesis of G Films Deposited on Quartz

Alginic acid (800 mg) from Aldrich was dissolved in a 10 mL aqueous solution. An amount of 1.6 mL of NH_4_OH solution (28–30% NH_3_ in water) was added to assist dissolution of alginic acid completely. After 2 h under magnetic stirring at room temperature, the solutions were filtered through a syringe of 0.45 µm pore size to remove the impurities present in the commercial alginic acid. The films were prepared on a previously cleaned quartz plate (2 × 2 cm^2^) by casting 500 µL of filtered solution at 4000 rpm in 45 s. Once dried on a hot plate, the films were pyrolyzed under Ar flow or Ar/H_2_ (5 vol%) or Ar/H_2_ (10 vol%) flow (200 mL min^−1^), increasing the temperature at a rate of 5 °C min^−1^ up to 900, 1000, 1100, and 1200 °C, respectively, holding the final temperature for 1 h. The sample was allowed to cool at room temperature under inert atmosphere by stopping electrical heating.

### 2.3. Characterization Techniques

Raman spectra were recorded at ambient temperature with 514 nm laser excitation on a Renishaw In Via Raman spectrometer (Renishaw, Gloucester, UK) equipped with a charge coupled device (CCD) detector. Transmission electron microscopy (TEM) images were recorded by using a Philips CM 300 FEG system with an operating voltage of 100 kV. AFM images were made with a Multimode Nanoscope 3A equipment (Nanoscope, Dallas, TX, US) working in tapping mode, using mica as substrate. X-ray photoelectron spectroscopy (XPS) were recorded on a SPECS spectrometer (SPECS, Berlin, Germany) equipped with a Phoibos 150 9MCD detector (SPECS, Berlin, Germany) using a non-monochromatic X-ray source (Al and Mg) operating at 200 W. The samples were evacuated in the prechamber of the spectrometer at 1 × 10^−9^ mbar. Some of the samples have been activated in situ in nitrogen flow at 450 °C for 3 h followed by evacuation at 10^−8^ mbar. The intensity ratios of components were obtained from the area of the corresponding peaks after nonlinear Shirley-type background subtraction and corrected by the transmission function of the spectrometer (SPECS, Berlin, Germany). Electrical conductivity measurements were performed using a potentiostat (Metrohm, Herisau, Switzerland) coupled with a four-tips head.

### 2.4. Photoelectronic Experiment

The experiments were carried out in a quartz cylindrical three-electrode cell on a Versastat electrochemical workstation with an (N)G or G photoelectrode as the working electrode, a platinum foil as the counter electrode and Ag/AgCl (saturated KCl) as the reference electrode. The working electrode was irradiated using a LED lamp as the light source through an optical fiber that indicates the photoelectrode from the front. Line-sweep voltammograms were measured at a scan rate of 50 mV/s in an aqueous solution of 1 M LiClO_4_ that had been previously purged with argon gas for 30 min. The photoelectric experiments were performed with a positive 1.1 V applied bias, and the production of H_2_ evolved in the photoreactor was determined by gas chromatography using GS-MOL column of 15 m and 0.55 mm ID with Thermal conductivity detector (TCD).

## 3. Results and Discussion

**Scheme 2:**
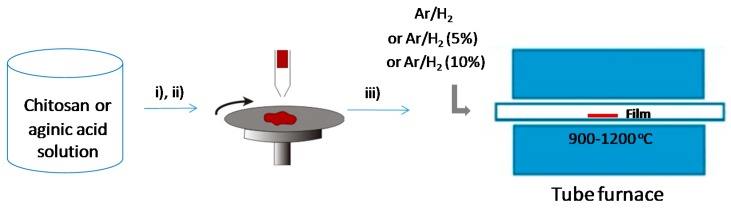
Steps in the preparation of the graphene or N-doped graphene film on quartz: (i) dissolution of chitosan or alginic acid in aqueous solution of acetic acid or ammonia; (ii) spin coating and water evaporation; (iii) pyrolysis of the sample at different temperatures (900–1200 °C) under Ar, or Ar/H_2_ (5%), or Ar/H_2_ (10%) flow.

Films of ammonium alginate and chitosan on clean quartz substrates were prepared by spin coating and their pyrolysis was carried out at temperatures in the range from 900 to 1200 °C either under a flow of Ar or under Ar containing 5 or 10 vol% H_2_. Scheme 2 shows the preparation process for the samples. Preliminary controls revealed that higher pyrolysis temperatures result in an almost complete volatilization of the biopolymer with no graphene material remaining, while higher H_2_ proportions do not apparently play any additional beneficial role in the quality of the resulting graphene. As commented above, the rationale was to increase the reductive conditions of the carbonization process, leading to a decrease of the residual oxygen content of the defective graphene films formed in the process by evolution of H_2_O or other gases.

To assess the influence of the pyrolysis temperature and presence of H_2_ and its concentration, the resulting films were characterized by Raman spectroscopy, XPS analysis, and electrical measurements and tested as electrodes for H_2_ evolution reaction. The morphology and thickness of the films were studied by AFM and scanning electron microscopy (SEM) of the films, as well as TEM of small pieces scratched from the films.

**Figure 1:**
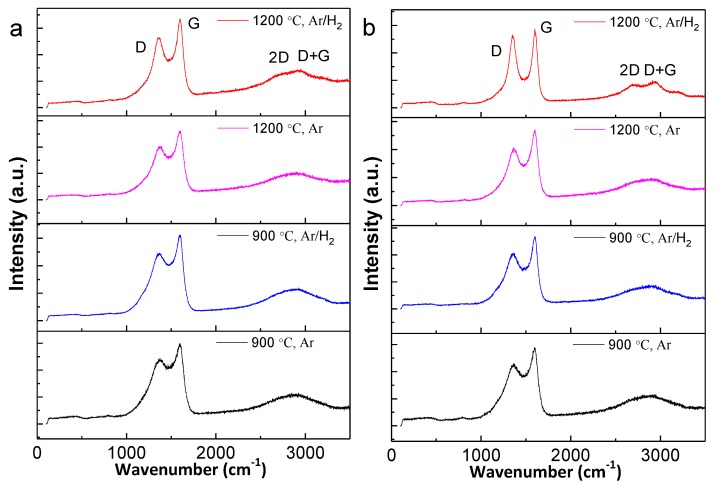
Raman spectra of (N)G prepared from chitosan (**a**) and G prepared from alginic acid (**b**) pyrolyzed at 900, 1200 °C under Ar or Ar/H_2_ (5%) flow, respectively.

As it was expected in view of the abundant literature data, Raman spectroscopy was a useful and convenient technique to follow the influence of the pyrolysis temperature and H_2_ concentration on the density of defects present on the resulting graphene film samples [19,20,21]. Figure 1 shows a set of Raman spectra to illustrate the type of changes observed in the characteristic graphene peaks by presenting two extreme cases, while Figures S1 and S2 include a complete set of Raman spectra for other films prepared in the present study.

There are several parameters used to assess the spectral changes occurring under the various preparation conditions, namely, the ratio between the intensity of the G vs. the D bands (*I*_G_/*I*_D_), the full width at half height of the G and D peaks (*fwhp*_G_ and *fwhp*_D_) and the resolution of the peaks measured by the ratio between the maximum intensity of the G band and the valley between the G and D bands (R). The higher the *I*_G_/*I*_D_ ratio, the narrower the G and D peaks and the higher the resolution between the peak and the valley, the higher was assumed to be the quality of the resulting graphene film, according to Raman spectroscopy. The corresponding values of the three parameters determined from the experimental Raman spectra are tabulated in Table 1.

As can be seen in Table 1 and also visually in Figure 1, most of the parameters determined by Raman indicate that the highest quality samples, both for alginic acid and chitosan, are achieved, when the pyrolysis was carried out at 1200 °C in the presence of 10% H_2_. As shown in Table 1, *I*_G_/*I*_D_ ratio is not the most useful parameter to follow the spectroscopic changes (see entries 2 and 3 in Table 1) that can be better assessed by *fwh*p of the G and D peaks and the resolution between peak and valley. It should be noted that the increase in the H_2_ content from 5 to 10% during the 12 h experiment does not substantially improve the Raman quality parameters (see Table 1, Figure S3), while it represents twice H_2_ consumption. On the other hand, temperatures higher than 1200 °C lead to the complete volatilization of the precursor without any residual graphene film remaining on the quartz plate and, therefore, it appears to be an upper limit on the temperature of the process.

It is particularly worth noting that the spectroscopic differences between alginic acid and chitosan probably reflect that in the last case, N atoms as dopant element are present on the resulting graphene ((N)G). The presence of dopant N heteroatoms should introduce some features (defects) in the Raman spectrum that limit possible decreases in the intensity or width, or both, of the D band. Accordingly, in the case of graphene derived from alginic acid, much sharper G and D bands were recorded, while the presence of the 2D and D + G overtones appeared also well resolved in the higher frequency region of the spectrum. The results presented in Figure 1, Figures S1–S3 and Table 1 clearly document the positive influence of optimization of pyrolysis temperature and the presence of H_2_ on the Raman spectra of the resulting graphene films. These spectral changes are associated to an improvement on the quality of the graphene films as consequence of their lower oxygen content.

The beneficial influence of the optimal pyrolysis temperature and the presence of H_2_ on the decrease on the oxygen content of the resulting films were also confirmed by XPS analysis. The carbon, oxygen, and nitrogen content for each of the films prepared starting from alginic acid and chitosan under the various preparation conditions are summarized in Table 2. As can be seen in Table 2, the oxygen content determined from the atomic percentage measured by XPS depends on the nature of the biopolymer, being higher for chitosan than for alginic acid, and decreases gradually with the pyrolysis temperature up to the 1200 °C, reaching the lowest values of 6.64, 6.92 at%, for G or (N)G, respectively, under a flow of Ar/H_2_.

The presence of H_2_ during the pyrolysis produces defective graphene samples wherein the oxygen content is consistently lower than that of the analogous samples prepared in the absence of H_2_. These analytical XPS data agree with the hypothesis that the H_2_ can react with the oxygen from the residual oxygenated functional groups during pyrolysis, resulting in the removal of some reactive oxygen atoms or functional groups from the resulting graphene samples. However, comparing with the samples obtained in the absence of H_2_, the difference in the oxygen content is minor, indicating that only a small amount of oxygen is eliminated due to H_2_. Also, the carbon content shows a trend with the pyrolysis temperature and becomes slightly higher by introducing H_2_ during the process. Thus, the carbon content of 79.68 at% from (N)G and 87.40 at% from G achieved at a pyrolysis temperature of 900 °C under Ar increased gradually to 92.22 and 93.36 at%, respectively, when the pyrolysis temperature was 1200 °C and H_2_ gas was present. As it is known, the carbon to oxygen atomic ratio (C/O ratio), which relates to the degree of oxidation, is one of the important factors determining the graphene quality. The highest C/O ratio for (N)G and G obtained at a pyrolysis temperature of 1200 °C under Ar/H_2_ is 13.33 and 14.06, respectively. These C/O ratios compare favorably with the values of 4.43 and 6.94 obtained at 900 °C under Ar.

It is worth noting that there was a jump of C/O ratio in G from 6.94 to 10.24 when H_2_ was introduced into the system at a pyrolysis temperature of 900 °C. It should also be noted that these values obtained at the high pyrolysis temperature of 1100 or 1200 °C, regardless whether H_2_ is present, are higher than most of the C/O ratios achieved for rGO prepared with different reduction methods [22,23,24,25]. For (N)G, the presence of H_2_ resulted in a slightly decreased nitrogen content in the samples, having a similar influence on this heteroatom as the increase in the pyrolysis temperature. This probably reflects the higher graphitization of carbon in these conditions. The highest N doping level of 2.32 at% was achieved at a pyrolysis temperature of 900 °C under Ar and then diminished to 2.06 at% in the presence of H_2_, which finally decreased to 0.86 at% at a pyrolysis temperature of 1200 °C under Ar/H_2_.

**Figure 2:**
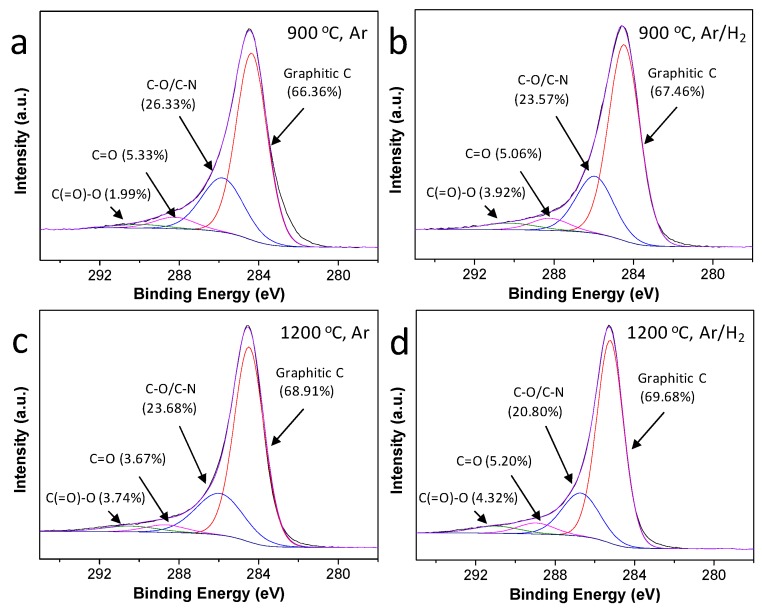
High resolution XPS of C 1s peak of (N)G pyrolyzed at 900 °C under Ar (**a**) or Ar/H_2_ (5%) (**b**) and pyrolyzed at 1200 °C under Ar (**c**) or Ar/H_2_ (5%) (**d**).

**Figure 3:**
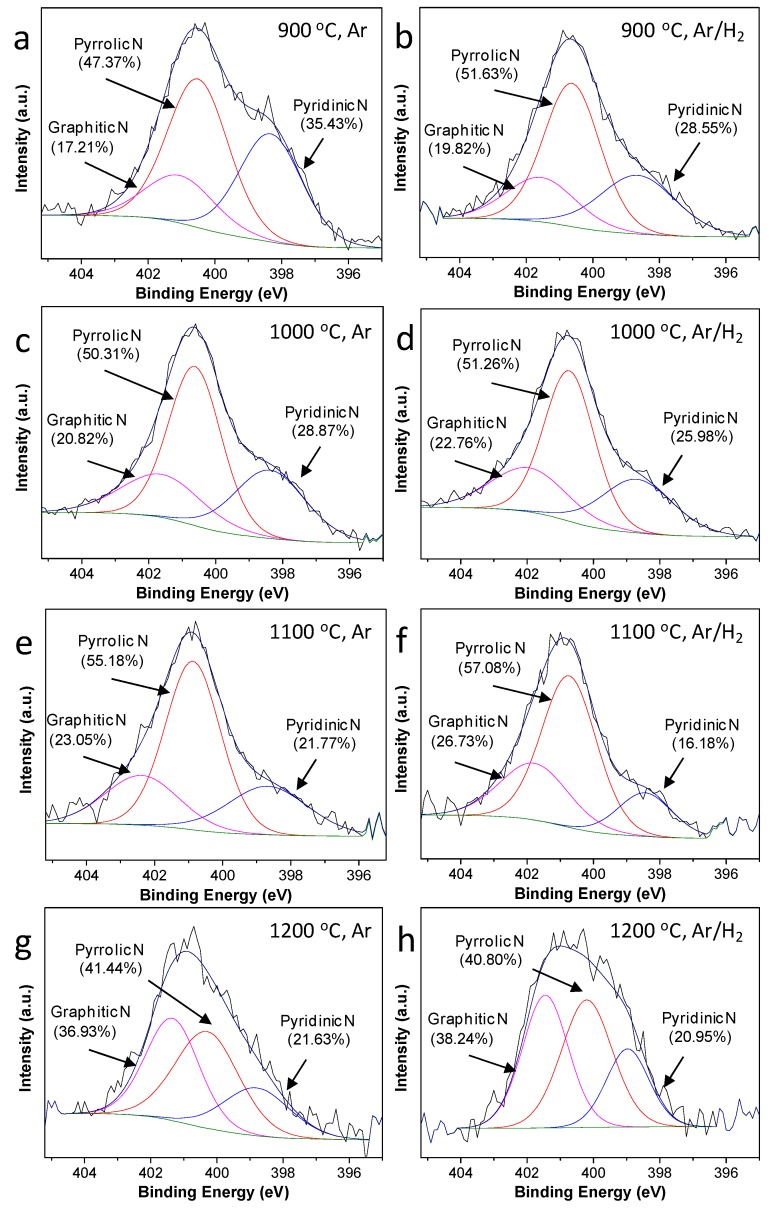
High resolution XPS of N1s peak of (N)G prepared at the pyrolysis temperature of 900 (**a**,**b**), 1000 (**c**,**d**), 1100 (**e**,**f**), 1200 (**g**,**h**) °C under Ar (left side) or Ar/H_2_ (5%) (right side), respectively.

Tables S1 and S2 show the detailed bonding information of C 1s for all the G samples as well as also that of N 1s for the (N)G. Some representative high-resolution XPS C 1s and N 1s peaks for (N)G samples are presented in Figure 2 and Figure 3, while some high-resolution XPS C 1s peaks for G samples are presented in Figure S4. In general, for the high-resolution XPS C 1s peak of all the graphene samples, the peaks can be deconvoluted into four individual components appearing at binding energy values 284.5, 285.9, 288.3, and 290.2 eV, which should correspond to graphitic C, C-O/C-N, C=O, and C(=O)-O, respectively. It seems that increasing pyrolysis temperature as well as the presence of H_2_ during the process have a positive influence on the quality of G and (N)G samples, resulting in the materials having slightly higher percentage of graphitic C and lower percentages of C-O/C-N and C=O bonds. Thus, it was observed that the C 1s peak becomes narrower with these two parameters. As an example, (N)G prepared at 1200 °C under Ar/H_2_ contains, according to the best fitting, a 69.68% of graphitic C, and a 20.80% of C-O/C-N, 5.20% of C=O, and 4.32% of C(=O)-O. In comparison, (N)G obtained at 900 °C under Ar contains a 66.36% of graphitic C, and percentages of 26.33, 5.33, and 1.99% of C-O/C-N, C=O, and C(=O)-O, respectively.

In contrast to the (N)G sample prepared at 900 °C under Ar, the XPS N 1s peak shown in Figure 3 can be fitted to three main peaks, centered at 398.5, 400.6, 401.6 eV, corresponding to pyridinic, pyrrolic, and graphitic N, respectively. The contents of these three N components are 35.43, 47.37, and 19.21%, respectively. This peak analysis indicates that the prevalent families of N atoms incorporated into the graphene are pyridinic and pyrrolic N.

As can be seen in Table 2 and Figure 3, with the increase of pyrolysis temperature, the general tendency observed is a gradual decrease in the percentage of pyridinic N, while the proportion of pyrrolic N is maintained and the contribution of graphitic N increases. This trend indicates that the thermodynamically more stable form of N atoms in (N)G is graphitic N, while that of pyridinic N appears to be the weakest, disappearing as the pyrolysis temperature increases. On the other hand, following a similar trend with the influence of pyrolysis temperature, the presence of H_2_ slightly decreases the N content and at the same time changes also the distribution of this element among the three major N families, increasing the contribution of graphitic N in the (N)G samples.

Since, as just commented, Raman spectroscopy and XPS analysis can report on the quality of the defective graphene samples as a function of the preparation conditions, based on the density of defects and oxygen content, additional experiments were carried out at 1100 °C using 10% H_2_ in the gas flow during the pyrolysis step. As indicated above, it seems that, in the presence of H_2_, the differences in the samples pyrolyzed at 1100 or 1200 °C are minimal, although the films become thinner when the concentration of H_2_ increased. It was observed that the samples prepared at 1100 °C under 10% H_2_ have almost identical Raman spectra and XPS data with the analogous samples prepared with 5% H_2_. These data indicate that 5% of H_2_ content during the pyrolysis step meets a compromise between better quality films and low H_2_ consumption.

**Figure 4:**
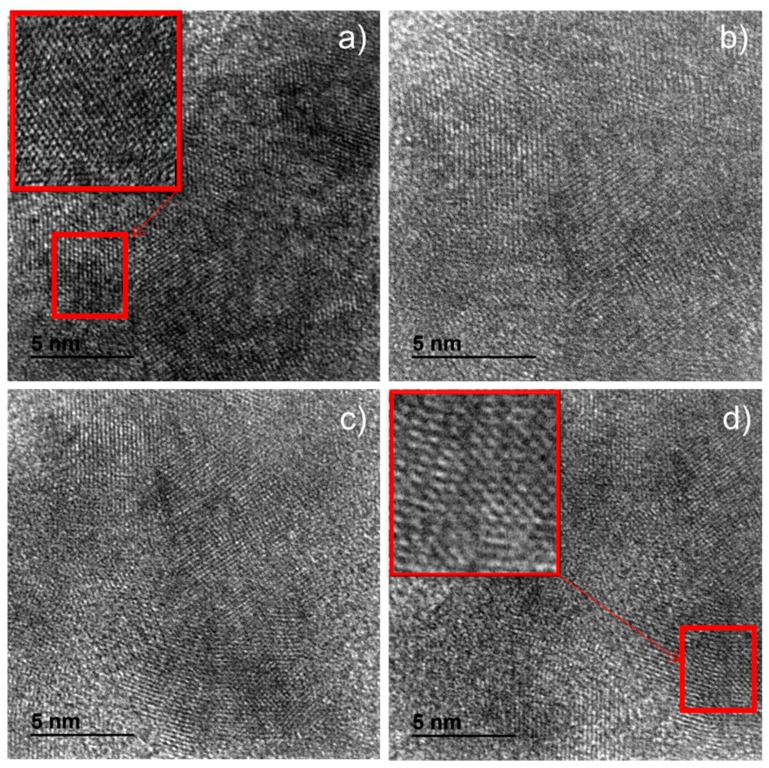
Set of TEM images taken for different four different parts (**a**–**d**) of (N)G pyrolyzed at 1200 °C under Ar/H_2_ (5%). The hexagonal arrangement observed in the high-resolution images has been highlighted in red. The inset shows the Fourier-transformed electron diffraction pattern of image (**d**).

The morphology of the defective films was studied by SEM and TEM and the thickness was determined by AFM. No obvious differences among the various films as a function of the pyrolysis temperature in the range of 900–1200 °C or prepared in the absence and presence of 5% H_2_ were observed by SEM. Nevertheless, the films prepared from chitosan appeared consistently in SEM with much lesser roughness than those from alginic acid. Perusal of the TEM images clearly indicates that the degree of ordering and graphitization of the films prepared at 1200 °C in the presence of H_2_ was higher than those obtained at 900 °C in its absence. Figure 4 shows representative TEM images and a Fourier-transformed electron diffraction pattern taken for (N)G pyrolyzed at 1200 °C under 5% H_2_ to illustrate the improvement in the crystallinity of the sheets upon optimization of the preparation conditions.

**Figure 5:**
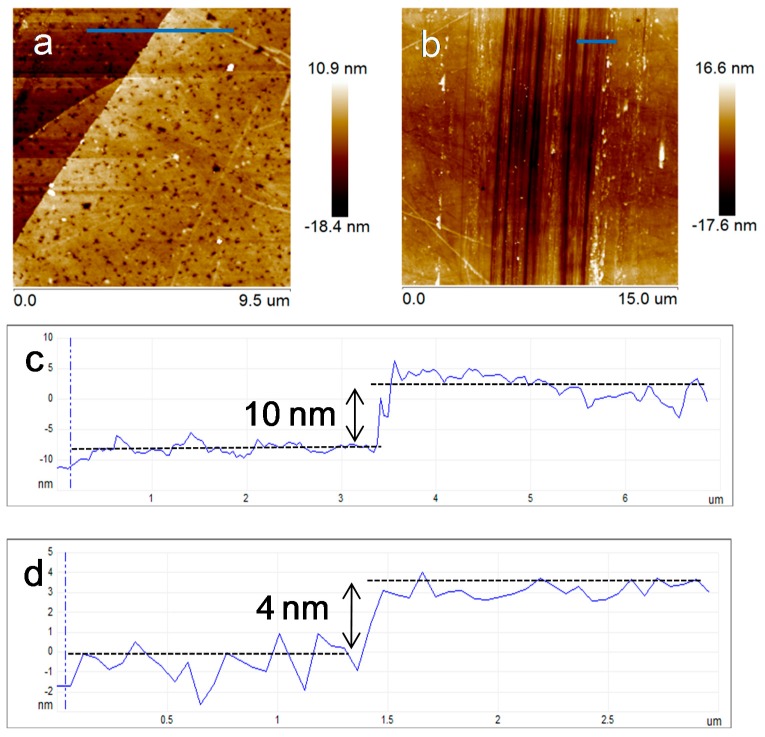
AFM images of two (N)G films prepared from chitosan pyrolyzed at 1100 (**a**) and 1200 °C under Ar/H_2_ (5%) flow (**b**). Plots **c** and **d** show the section profiles of images (**a**–**b**), respectively, along the blue lines indicated in the frames (**a**–**b**). Thicknesses about 10 and 4 nm were determined for the films prepared at 1100 and 1200 °C, respectively.

AFM measurements indicate that starting from alginic acid or chitosan films of the same thickness, the resulting graphene film becomes thinner upon increasing the pyrolysis temperature and when H_2_ was present in the atmosphere during the process. As an example, Figure 5 presents representative AFM images and the corresponding film thicknesses for two (N)G films prepared under different conditions.

**Figure 6:**
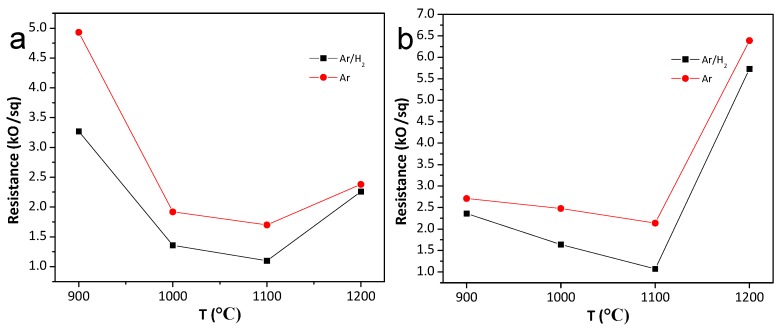
Plots of the electrical resistance for (N)G (**a**) and G (**b**) films on quartz substrate prepared at different pyrolysis temperatures under Ar or Ar/H_2_ (5%) flow.

As commented at the beginning of the manuscript, the purpose of the present study was to determine if there is an increase in the conductivity of the graphene films upon optimization of the preparation conditions and, particularly, by optimization of the pyrolysis temperature and introduction of H_2_ in the gas flow. The results of the conductivity measurement are presented in Figure 6, where plots of the electrical conductivity measured using a four-tips head vs. the pyrolysis temperature in the absence and presence of H_2_ are presented. As one can see in Figure 6, the electrical resistance of the defective graphene films prepared from alginic acid and chitosan follows a similar trend with the pyrolysis temperature and in the presence of H_2_ although it was somewhat more remarkable in the case of chitosan. The electrical resistance of the defective graphene films decreases with the temperature from 900 to 1100 °C and it increases beyond this temperature. The presence of 5% H_2_ during the pyrolysis has a beneficial influence, decreasing the electrical resistance at all temperatures and for both precursors. By optimizing the pyrolysis conditions, the electrical resistance could be decreased by a factor of about 3 and 5 for alginic acid and chitosan, respectively. The minimum electrical resistance measured in the present study was 1000 and 1100 Ω/sq for defective films from alginic acid and chitosan, respectively.

The most probable reason why the electrical resistance increases at high temperature is the inappropriateness of the four-tips head to measure the electrical conductivity of the thinnest films of just a few nanometers thickness that are obtained at 1200 °C. It may also happen that these films are also not totally continuous at the millimetric length scale needed for the electrical resistance measurements due to volatilization of the biopolymer.

**Figure 7:**
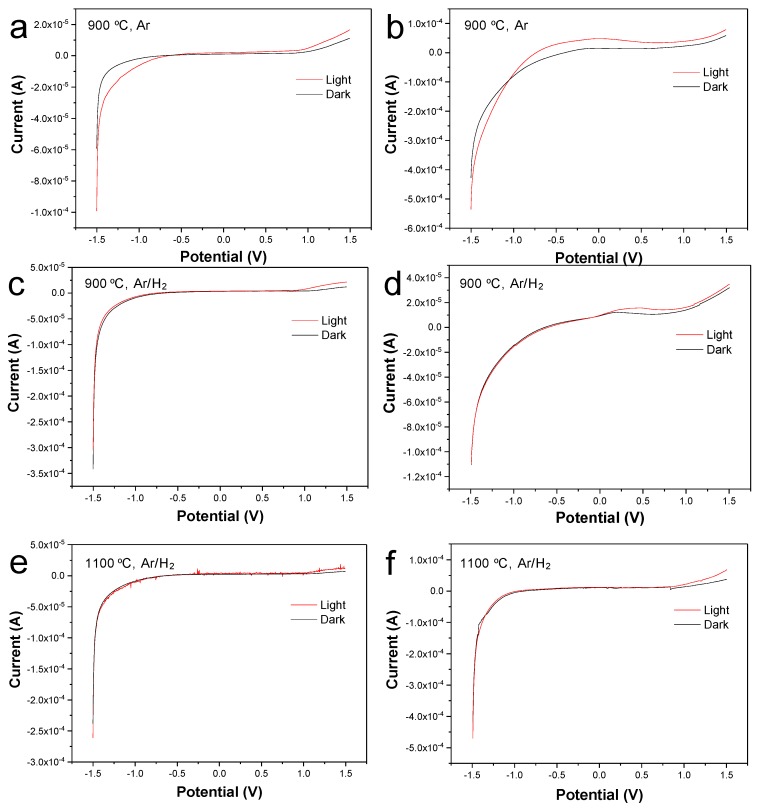
Line-sweep voltammogram (LSV) curves measured at a scan rate of 50 mV/s in darkness or under LED illumination from the electrodes made of (N)G prepared at 900 °C under Ar (**a**) or Ar/H_2_ (**c**), at 1100 °C under Ar/H_2_ (**e**) or G prepared at 900 °C under Ar (**b**) or Ar/H_2_ (**d**), at 1100 °C under Ar/H_2_ (**f**), respectively.

The (N)G and G films were used as electrodes for hydrogen evolution reaction. A similar test was carried out in previous work from the group, using (N)G film as the working electrode and aqueous Na_2_S/Na_2_SO_3_ solution as electrolyte under illumination of a UV/Vis 300 W Xe lamp [26]. In the present work, line-sweep voltammograms (LSV) were performed with the graphene electrodes at a scan rate of 50 mV/s in darkness or under LED lighting, using a 1 M LiClO_4_ aqueous solution as electrolyte. The results are shown in Figure 7. As we can see, for both (N)G and G, the film prepared at 1100 °C, Ar/H_2_ flow shows a smaller difference in the current density between light and dark, comparing with the film prepared at 900 °C under Ar/H_2_ or Ar flow. Since the defective graphenes with bandgap are able to photogenerate charges under light irradiation, making the current a little higher than the value in darkness, the degree of the difference between these two curves can somewhat indicate the defective level of the films. These results from the experimental samples demonstrate that the film prepared at higher pyrolysis temperature and in the presence of H_2_ shows a more perfect graphene structure with lower density of defects, which is in good agreement with the characterization results from Raman, XPS, and TEM.

**Figure 8:**
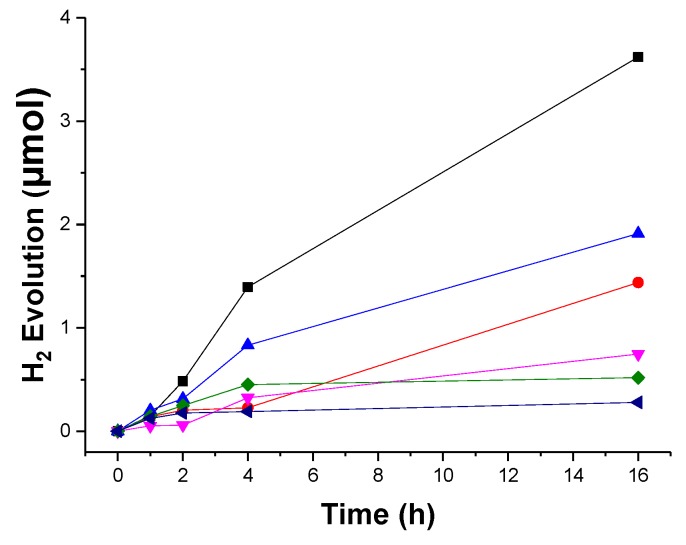
H_2_ evolution obtained in darkness or under LED illumination using the electrodes made of (N)G or G film prepared at 900 or 1100 °C under Ar or Ar/H_2_ flow: (◀) (N)G, 900 °C, Ar, Light; (♦) (N)G, 1100 °C, Ar/H_2_, Light; (▼) G, 900 °C, Ar, Light; (●) G, 1100 °C, Ar/H_2_, Dark; (▲) G, 900 °C, Ar/H_2_, Light; (■) G, 1100 °C, Ar/H_2_, Light.

**Scheme 3:**
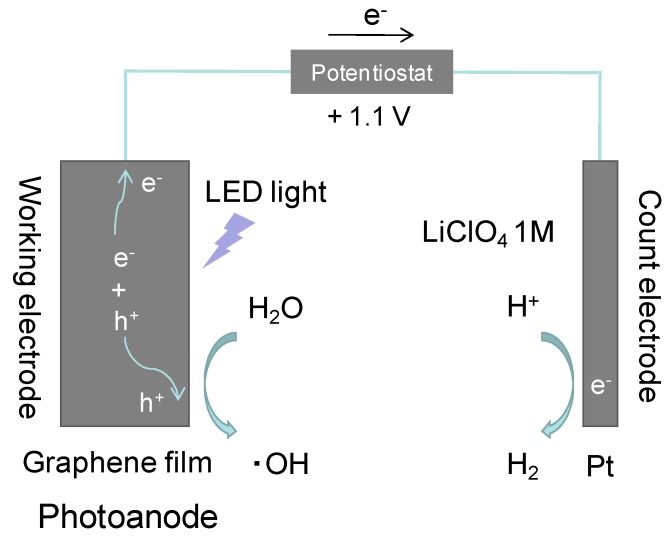
Possible mechanism of photoelectrocatalysis for the graphene film used as electrode under LED illumination with a positive 1.1 V applied bias. The test was performed using an aqueous solution of 1 M LiClO_4_ as electrolyte, a Pt foil as counter electrode, and Ag/AgCl as reference electrode.

Also, the production of H_2_ under light or dark condition was measured during the photoelectric test with an applied bias of +1.1 V. The results are presented in Figure 8. As it can be seen, G films exhibit better photocatalytic activity than (N)G films, although the activities of both two types of films are very low, in agreement with the composition of the carbonaceous materials and the absence of any sacrificial reagent. The most perfect film, G film prepared at 1100 °C under Ar/H_2_, shows the best catalytic activity, achieving a H_2_ production of 3.62 µmol after 16 h under LED illumination, while the values for G films pyrolyzed at 900 °C under Ar or Ar/H_2_ condition are 0.75 and 1.91 µmol, respectively. Control experiment for the G film prepared at 1100 °C under Ar/H_2_ was performed under dark condition and the sample exhibited lower activity, with a H_2_ yield of 1.44 µmol. In the case of N-doped graphene, although the activity for the experimental films is very low even under LED light irradiation, it still could be seen that the film prepared at 1100 °C under Ar/H_2_ shows slightly higher activity than the film prepared at 900 °C under Ar. The more efficient performance of the graphene film should be due to the decrease in the intensity of defects on the structure, resulting from the higher pyrolysis temperature and the presence of H_2_. Similar with the photoelectrocatalysis based on other typical semiconductors [27], a proposed mechanism for the reaction using the graphene film as electrode is presented in Scheme 3. The electrode made of the graphene film is supposed to act as photoanode, where the photogenerated charges can be separated efficiently with the external bias of +1.1 V.

## 4. Conclusions

The present study has shown that the presence of a small percentage of H_2_ during the pyrolysis of natural filmogenic polysaccharides decreases the density of defects on the resulting few-layers undoped and doped graphenes, although it was not possible to remove completely the presence of oxygen in the samples, even at the highest pyrolysis temperatures. This incomplete decrease of the oxygen functionalities is reflected in an improvement of the electrical conductivity of the samples that can reach a value of 1000 Ω/sq by pyrolysing alginic acid at 1100 °C under 5% H_2_. The electrode made of less defective, few-layers graphene film exhibits photoelectrocatalytic activity for H_2_ evolution at a positive applied bias under LED illumination. In the context of valorization of biomass waste, the present study shows that the properties of graphenes obtained from these residues can be easily improved by adequate optimization of the pyrolysis conditions, the challenge of complete oxygen removal still remaining.

## Figures and Tables

**Table 1:** Parameters determined from the Raman spectra to assess the influence of the pyrolysis temperature and H_2_ concentration on the defect density on the resulting graphene films ^1^.

Entry	T (°C)	Gas	(N)G				G			
			*I*_G_/*I*_D_	*fwhp*_G_	*fwhp*_D_	*R*	*I*_G_/*I*_D_	*fwhp*_G_	*fwhp*_D_	*R*
1	900	Ar	1.26	176.2	331.0	1.57	1.26	155.3	330.1	1.70
2	900	Ar/H_2_ (5%)	1.27	156.6	291.9	1.68	1.31	114.2	275.9	2.03
3	1000	Ar	1.26	161.6	304.2	1.75	1.31	141.5	301.9	1.82
4	1000	Ar/H_2_ (5%)	1.26	149.4	247.1	1.76	1.28	113.6	254.0	2.07
5	1100	Ar	1.30	144.0	307.1	1.76	1.28	135.9	257.3	1.93
6	1100	Ar/H_2_ (5%)	1.29	119.0	214.3	2.03	1.26	105.2	216.9	2.31
7	1100	Ar/H_2_ 10%)	1.29	115.2	208.1	2.07	1.29	98.9	208.5	2.36
8	1200	Ar	1.31	138.2	296.2	1.79	1.30	120.2	221.2	2.07
9	1200	Ar/H_2_ (5%)	1.31	116.5	214.6	2.15	1.08	89.6	122.4	3.18
10	1200	Ar/H_2_ 10%)	1.31	112.6	216.8	2.21	1.13	87.4	121.4	3.22

^1^*fwh*p: full width at half peak height; *R*: resolution between the G and the D peak. The gray cells highlight the best values according to each quality indicator.

**Table 2:** Elemental composition of graphene films prepared from chitosan or alginic acid according to the XPS data ^1^.

Entry	T (°C)	Gas	(N)G				G		
			C (at%)	O (at%)	N (at%)	C/O Ratio	C (at%)	O (at%)	C/O Ratio
1	900	Ar	79.68	18.00	2.32	4.43	87.40	12.60	6.94
2	900	Ar/H_2_	84.49	13.46	2.06	6.28	91.10	8.90	10.24
3	1000	Ar	87.79	10.17	2.03	8.631	91.50	8.50	10.76
4	1000	Ar/H_2_	90.70	7.45	1.86	12.17	92.18	7.82	11.79
5	1100	Ar	89.67	8.51	1.81	10.54	91.53	8.47	10.81
6	1100	Ar/H_2_	91.55	7.09	1.35	12.91	92.26	7.74	11.92
7	1200	Ar	91.41	7.44	1.16	12.29	92.06	8.05	11.59
8	1200	Ar/H_2_	92.22	6.92	0.86	13.33	93.36	6.64	14.06

^1^ The gray cells highlight the best values according to each quality indicator.

## Supplementary Materials

The following are available online at https://www.mdpi.com/2079-4991/9/6/895/s1, Figure S1: Raman spectra of (N)G prepared from chitosan pyrolyzed at 900, 1000, 1100, 1200 °C under Ar (a) or Ar/H_2_ (5%) (b) flow respectively. Figure S2: Raman spectra of G prepared from alginate pyrolyzed at 900, 1000, 1100, 1200 °C under Ar (a) or Ar/H_2_ (5%) (b) flow respectively. Figure S3: Raman spectra of (N)G prepared from chitosan (a) and G prepared from alginate (b) pyrolyzed at 1100, 1200 °C under Ar/H_2_ (5%) or (10%) flow respectively. Table S1: Distribution of C atoms among different chemical environments as determined by deconvolution of the high resolution XPS C 1s peak for all samples under study. Table S2: Distribution of N atoms among different chemical environments as determined by deconvolution of the high resolution XPS N 1s peak for the (N)G samples under study. Figure S4: High resolution XPS of C1s peak of G pyrolyzed at 900 °C under Ar (a) or Ar/H_2_ (5%) (b) and G pyrolyzed at 1200 °C under Ar (c) or Ar/H_2_ (5%) (d).
