# Supplementary material for: Quality Improvement of Few-Layers Defective Graphene from Biomass and Application for H2 Generation
Source: Nanomaterials (Basel). 2019 Jun 19;9(6):895. doi: 10.3390/nano9060895 (PMC6632024; doi:10.3390/nano9060895)
Supplement: Supplementary file 1 [file nanomaterials-09-00895-s001.pdf]

nanomaterials

MDPI

Supplementary Materials

Quality Improvement of Few-Layers Defective Graphene from Biomass and Application for H_2 Generation

Jinbao He, Aicha Anouar, Ana Primo * and Hermenegildo García *

Instituto Universitario de Tecnología Química, Consejo Superior de Investigaciones Científicas-Universitat Politècnica de Valencia, Av. De los Naranjos s/n, 46022 Valencia, Spain; 2131342@mail.dhu.edu.cn (J.H.); aian1@doctor.upv.es (A.A.)

* Correspondence: hgarcia@qim.upv.es (A.P.); aprimoar@itq.upv.es (H.G.); Tel.: +34-96-387-7800 (A.P.); Tel.: +34-96-387-7807 (H.G.)

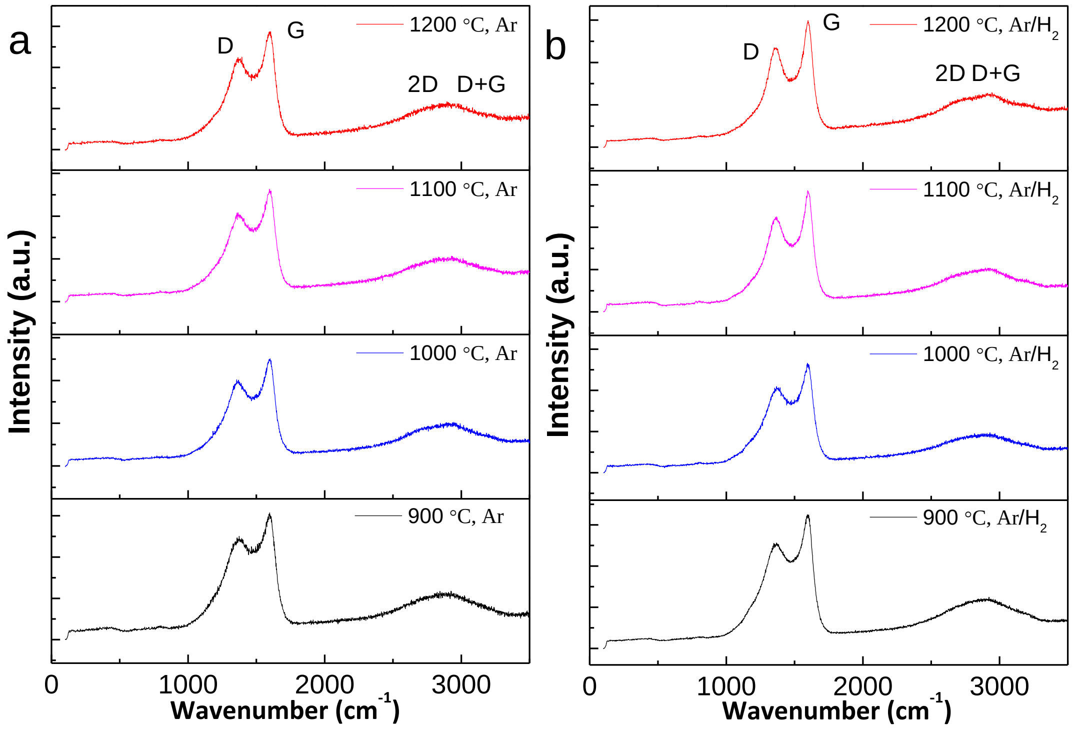

Figure S1. Raman spectra of (N)G prepared from chitosan pyrolyzed at 900, 1000, 1100, 1200 °C under Ar (**a**) or Ar/H_2 (5%) (**b**) flow respectively.

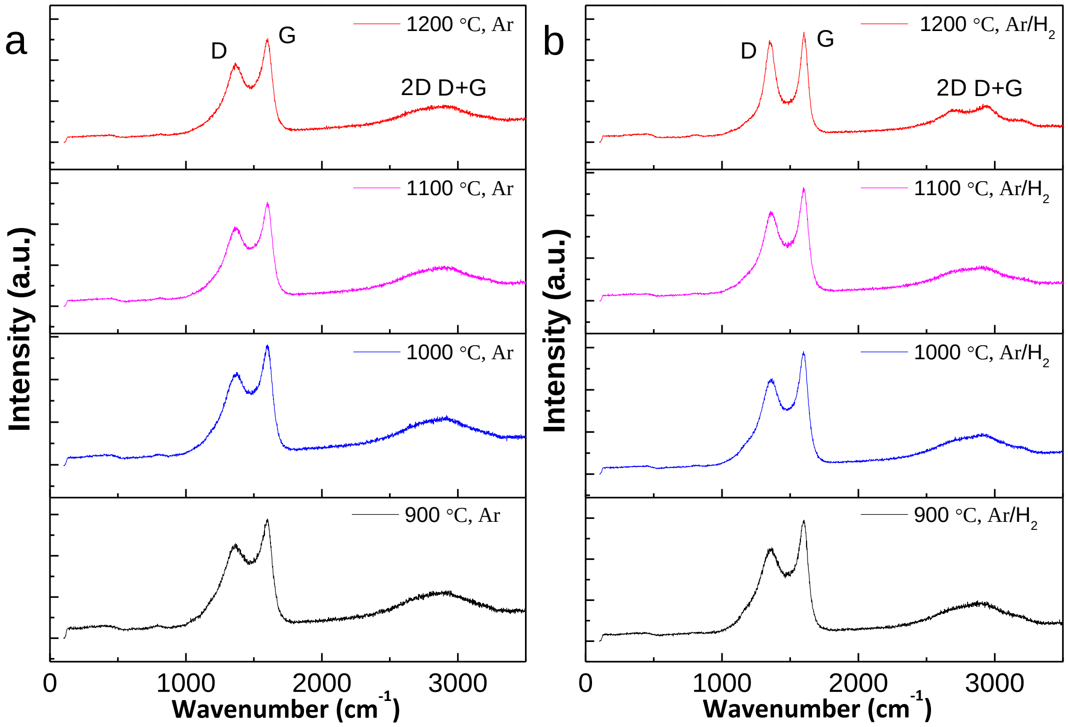

Figure S2. Raman spectra of G prepared from alginate pyrolyzed at 900, 1000, 1100, 1200 °C under Ar (**a**) or Ar/H_2 (5%) (**b**) flow respectively.

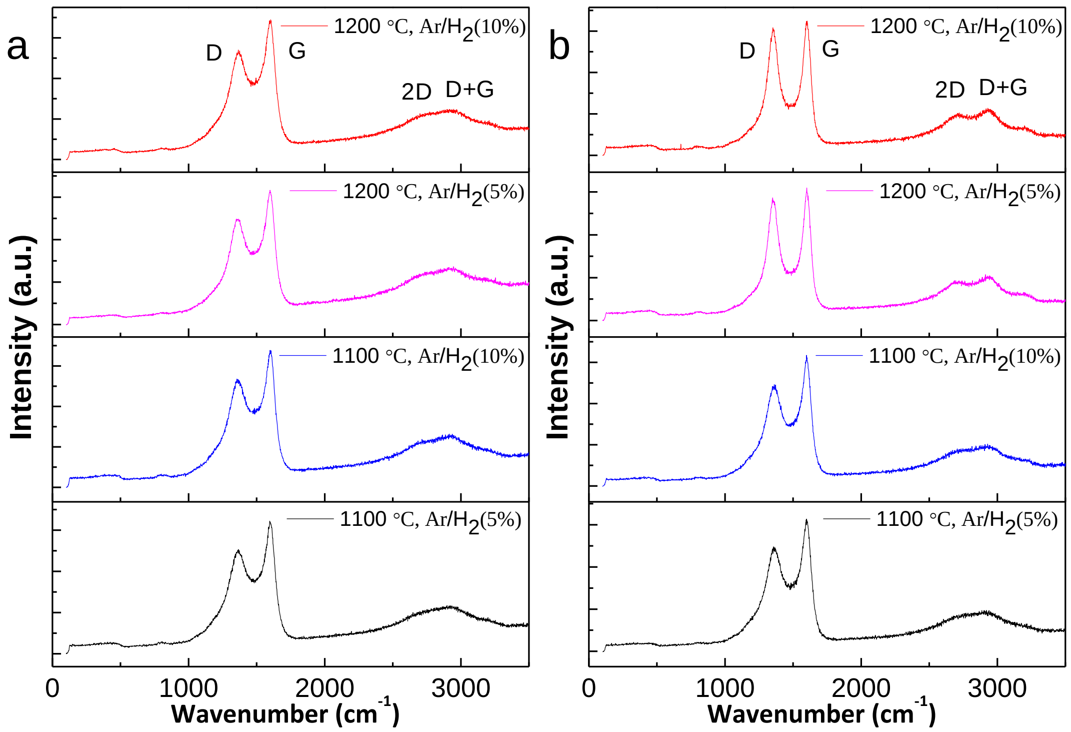

Figure S3. Raman spectra of (N)G prepared from chitosan (**a**) and G prepared from alginate (**b**) pyrolyzed at 1100, 1200 °C under Ar/H_2 (5%) or (10%) flow respectively.

Table S1. Distribution of C atoms among different chemical environments as determined by deconvolution of the high resolution XPS C 1s peak for all samples under study.

			(N)G				G			
Entry	T (°C)	Gas	C-C	C-O/C-N	C=O	O-C=O	C-C	C-O	C=O	O-C=O
1	900	Ar	66.36	26.33	5.33	1.99	63.91	25.73	6.20	4.16
2	900	Ar/H_2	67.46	23.57	5.06	3.92	66.25	22.95	5.20	5.60
3	1000	Ar	67.17	23.46	4.09	5.28	67.10	22.23	5.85	4.82
4	1000	Ar/H_2	68.14	23.84	3.30	4.72	67.35	23.13	3.43	6.09
5	1100	Ar	68.19	22.53	4.63	4.66	67.62	24.12	3.35	4.91
6	1100	Ar/H_2	68.60	22.81	2.81	5.78	67.67	22.63	4.23	5.47
7	1200	Ar	68.91	23.68	3.67	3.74	68.44	22.05	3.83	5.67
8	1200	Ar/H_2	69.68	20.80	5.20	4.32	70.44	21.39	3.61	4.57

Table S2. Distribution of N atoms among different chemical environments as determined by deconvolution of the high resolution XPS N 1s peak for the (N)G samples under study.

			(N)G		
Entry	T (°C)	Gas	Graphitic	Pyrrolic	Pyridinic
1	900	Ar	17.21	47.37	35.43
2	900	Ar/H_2	19.82	51.63	28.55
3	1000	Ar	20.82	50.31	28.87
4	1000	Ar/H_2	22.76	51.26	25.98
5	1100	Ar	23.05	55.18	21.77
6	1100	Ar/H_2	26.73	57.08	16.18
7	1200	Ar	36.93	41.44	21.63
8	1200	Ar/H_2	38.24	40.80	20.95

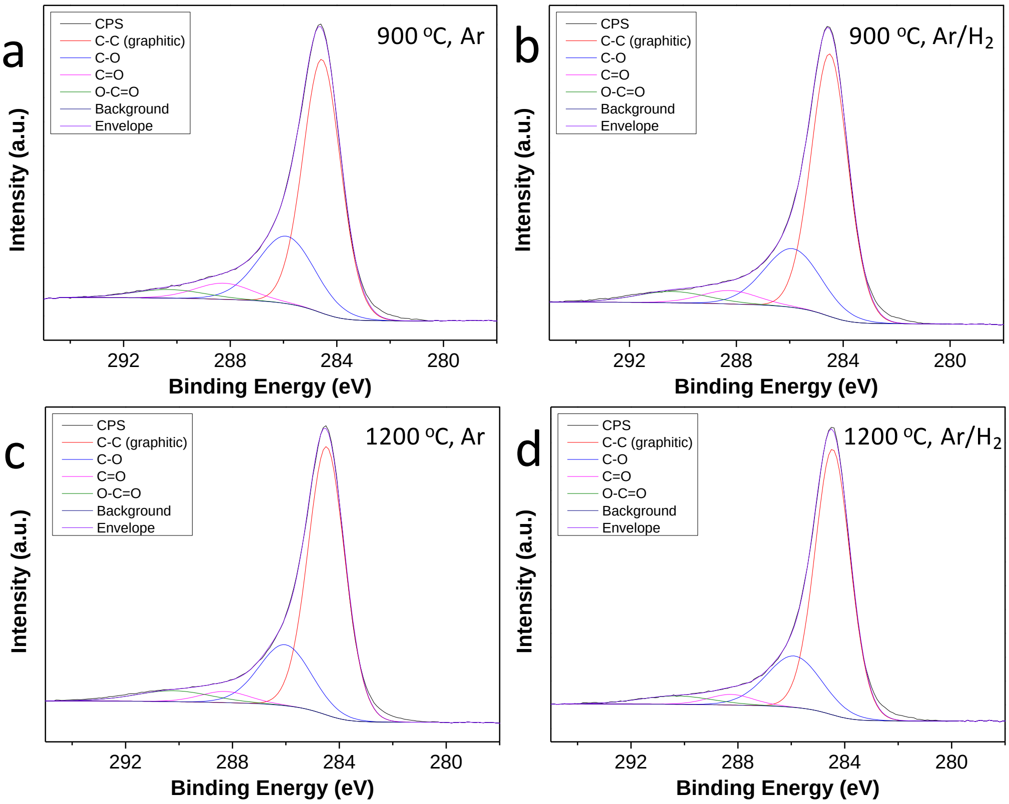

Figure S4. High resolution XPS of C1s peak of G pyrolyzed at 900 °C under Ar (**a**) or Ar/H_2 (5%) (**b**) and G pyrolyzed at 1200 °C under Ar (**c**) or Ar/H_2 (5%) (**d**).
